# The aging paradox of Cushing's syndrome: Stress without clear senescence?

**DOI:** 10.1111/jne.70213

**Published:** 2026-06-11

**Authors:** Israel Nunes Silveira, Alexandro Guterres

**Affiliations:** ^1^ Department of Genetics Ribeirão Preto Medical School, University of São Paulo – USP São Paulo Brazil

**Keywords:** biological aging, Cushing's syndrome, epigenetic aging, hypercortisolism, telomere dynamics

## Abstract

Cushing's syndrome (CS), characterized by chronic endogenous hypercortisolism and disruption of the normal circadian cortisol rhythm, represents a unique human model for investigating the interplay between stress endocrinology and biological aging. Glucocorticoid excess drives multiple convergent aging pathways: it suppresses telomerase activity and may accelerate telomere attrition, induces durable epigenetic remodeling, including hypomethylation of stress‐responsive genes such as *FKBP5*, TET‐mediated DNA hydroxymethylation changes, and broad dysregulation of circadian clock *loci*, and promotes telomere‐independent senescence through mitochondrial dysfunction, oxidative stress, and cytokine‐driven inflammation. These observations underpin a proposed dual‐pathway model of glucocorticoid‐accelerated aging, in which direct telomerase suppression and indirect epigenomic remodeling converge on common hallmarks of cellular senescence. However, clinical evidence remains heterogeneous and inconclusive: studies of leukocyte telomere length in CS report discrepant results attributable to assay platform differences, failure to correct for glucocorticoid‐induced lymphopenia, small sample sizes, and predominant reliance on cross‐sectional designs. Epigenetic clocks capable of quantifying biological age acceleration, particularly second‐generation tools such as GrimAge and DunedinPACE, have not yet been applied to CS cohorts, representing a critical evidence gap. Furthermore, virtually all molecular aging measurements in CS have been performed in peripheral blood, leaving the causal chain from glucocorticoid excess to organ‐level aging and excess mortality empirically unproven. Rather than a definitive null result, this paradox reveals that CS is an underexplored natural experiment: the syndrome's defined onset, quantifiable cortisol exposure, and surgically curable course offer a unique before‐after design for testing whether stress‐induced endocrine disruption genuinely accelerates biological aging through telomere and epigenetic mechanisms.

## 1 INTRODUCTION

### 1.1 Cushing's syndrome: Clinical burden, morbidity and mortality

Cushing's syndrome (CS) is characterized by chronic endogenous hypercortisolism arising from distinct etiological sources. Pituitary‐dependent CS, or Cushing's disease, results from ACTH hypersecretion by a pituitary adenoma and accounts for approximately 70% of all endogenous CS cases.^1^, ^2^ Ectopic ACTH syndrome, in which non‐pituitary tumors autonomously secrete ACTH, constitutes a smaller but clinically relevant subset of ACTH‐dependent hypercortisolism.^3^, ^4^ ACTH‐independent forms arise from primary adrenocortical tumors, adenomas or carcinomas, and complete the etiological spectrum of endogenous CS.^2^, ^4^ Regardless of etiology, prolonged exposure to supraphysiological glucocorticoid levels produces a characteristic clinical syndrome encompassing central obesity, hypertension, diabetes mellitus, dyslipidemia, osteoporosis, sarcopenia, immune suppression, and neuropsychiatric disturbances.^1^

The clinical burden of CS extends well beyond its symptomatic features: endogenous hypercortisolism is associated with markedly elevated morbidity and reduced life expectancy.^1^ Meta‐analyses and large national cohort studies report persistently increased overall and cardiovascular mortality in CS, a risk that remains elevated even after biochemical remission in many series.^5^, ^6^ Several cohort analyses corroborate that mortality during active disease is substantially higher and that excess risk may not be fully normalized after cure, implying durable consequences of prior hypercortisolism.^7^ The multisystem comorbidity profile of CS, including cognitive decline, hippocampal volume loss, accelerated atherosclerosis, and premature frailty, recapitulates features commonly associated with biological aging,^8^ raising the question of whether chronic glucocorticoid excess genuinely accelerates the molecular aging process or merely produces an aging‐like phenotype through independent pathways.

### 1.2 Molecular hallmarks of aging and the role of glucocorticoid excess

Aging is a progressive biological process characterized by the decline of cellular homeostasis, accumulation of molecular damage, and increased susceptibility to chronic disease and mortality.^9^, ^10^ Among the hallmarks of aging, telomere attrition and alterations in DNA methylation have emerged as robust molecular biomarkers of biological age. Although many studies associate shorter telomeres or epigenetic age acceleration with reduced lifespan, the strength and consistency of these associations vary considerably across populations and disease states.^9^, ^10^ Recent reviews have emphasized that, while telomere shortening is a consistent biological finding, its predictive value for mortality and age‐related outcomes remains modest and highly dependent on methodological factors such as assay type, tissue specificity, and population characteristics.^11^ Conversely, methylation‐based telomere length estimators (DNAmTL) have demonstrated stronger prediction of all‐cause mortality, frailty, and cancer risk compared to direct telomere length measures in large human cohorts, underscoring the value of integrating methylation and telomere dynamics as complementary biomarkers.^12^

The variability of these aging markers across populations suggests that additional upstream modulators must shape how they reflect the aging process. One of the most compelling candidates is the biology of chronic stress and endocrine dysregulation.^13^ Chronic activation of the hypothalamic–pituitary–adrenal (HPA) axis results in sustained glucocorticoid exposure, a central mediator of the physiological stress response.^14^ Glucocorticoids exert pleiotropic effects on cellular homeostasis: they can directly interfere with telomere maintenance by suppressing telomerase activity, and they also induce lasting epigenetic remodeling, including DNA methylation changes in stress‐responsive genes such as *FKBP5* and potentially in subtelomeric regions that regulate telomere stability.^15^ Importantly, glucocorticoid excess does not act exclusively through telomeric pathways: it also drives mitochondrial dysfunction, oxidative stress, disrupted autophagy, and cytokine‐mediated senescence, converging on multiple hallmarks of biological aging independently of telomere attrition.^16^, ^17^

### 1.3 Cushing's syndrome as a natural model of stress‐induced aging

**FIGURE 1:**
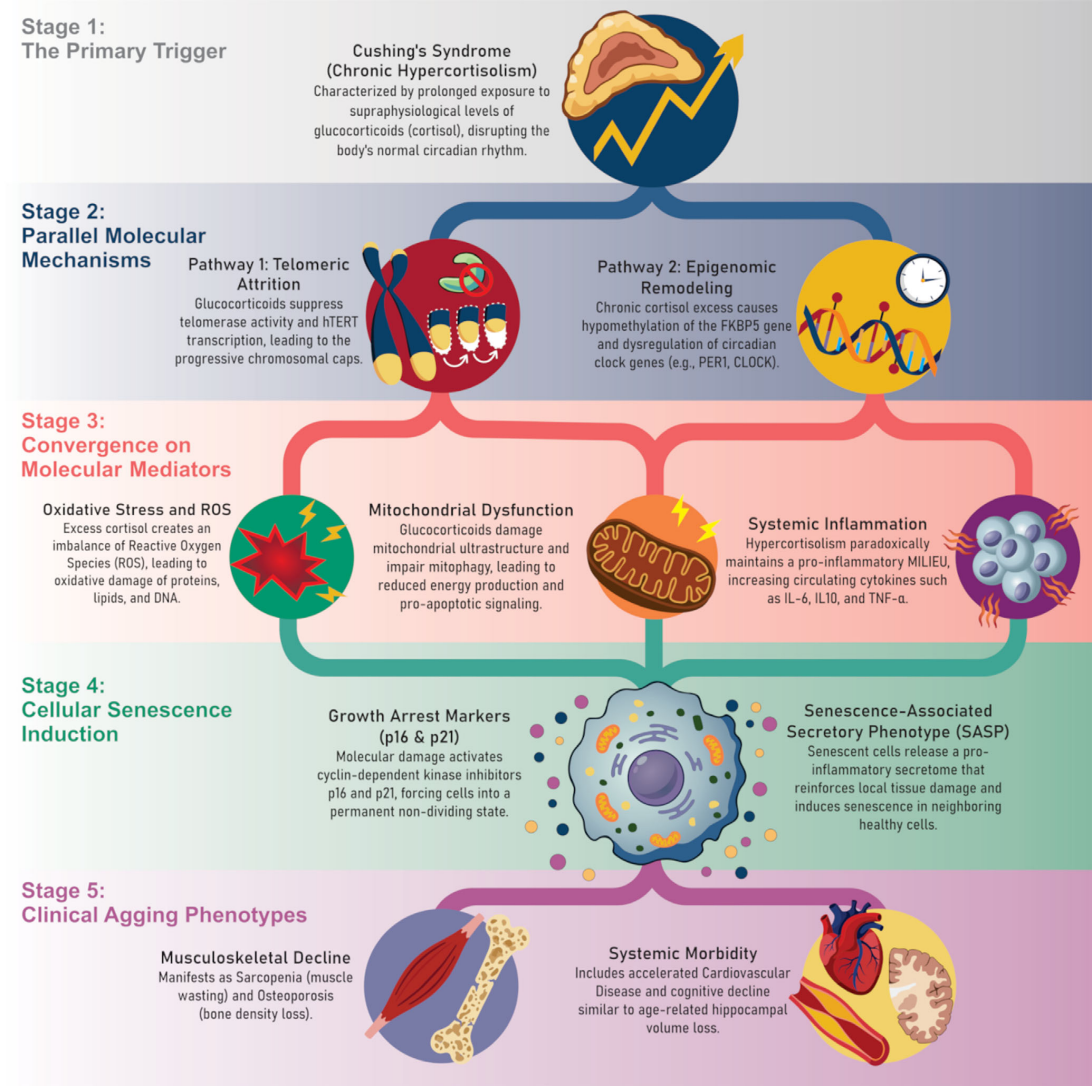
Dual‐pathway model of glucocorticoid‐driven biological aging in Cushing's syndrome. Schematic representation of the convergent mechanisms by which chronic endogenous hypercortisolism accelerates biological aging. (Left) The Telomeric Pathway: Glucocorticoid (GC) excess directly suppresses telomerase activity by inhibiting the transcription of the catalytic subunit *hTERT*, leading to progressive telomere attrition. (Right) The Epigenomic Pathway: GCs induce durable epigenetic remodeling, characterized by the hypomethylation of the *FKBP5* gene and the dysregulation of core circadian clock genes (*PER1‐3*, *CLOCK*), which disrupts cellular homeostasis and immune cycling. (Center) Mechanistic Convergence: Both pathways converge on telomere‐independent mediators, including increased mitochondrial reactive oxygen species (ROS) production, mitochondrial dysfunction, and a pro‐inflammatory milieu (elevated IL‐6, TNF‐α). This cumulative molecular damage triggers cellular senescence, marked by the upregulation of p16^INK4a^ and p21^CIP1^ and the development of a Senescence‐Associated Secretory Phenotype (SASP). Collectively, these processes drive the clinical aging phenotypes characteristic of Cushing's syndrome, such as sarcopenia, osteoporosis, accelerated atherosclerosis, and cognitive decline. CS, Cushing's syndrome; FKBP5, FK506‐binding protein 51; GCs, glucocorticoids; hTERT, human telomerase reverse transcriptase; IL‐6, interleukin‐6; PER/CLOCK, period/circadian locomotor output cycles kaput (circadian clock genes); ROS, reactive oxygen species; SASP, senescence‐associated secretory phenotype; TNF‐α, tumor necrosis factor‐alpha.

Cushing's syndrome, by virtue of its chronic and measurable endogenous hypercortisolism, therefore represents a unique natural model in which telomere attrition, epigenetic remodeling, and glucocorticoid excess can be examined concurrently and in real time.^1^ Rather than assuming their convergence, this clinical context provides the opportunity to formally test whether stress‐related endocrine disruption truly accelerates biological aging through a dual mechanistic pathway, direct suppression of telomere maintenance and indirect epigenetic modulation, or whether the relationship is more complex and context‐dependent.^15^, ^18^, ^19^ This dual‐pathway framework is summarized in Figure 1.

Under such a model, patients with CS would be expected to exhibit measurable acceleration of biological aging, including shorter telomeres, epigenetic age acceleration, and reduced life expectancy, particularly during active disease, with at least partial recovery after remission. Critically, the diversity of CS etiological subtypes, which differ in the tempo, severity, and circadian pattern of cortisol excess, provides an additional dimension for testing whether the magnitude of molecular aging acceleration is proportional to the nature of hypercortisolism.^1^, ^2^, ^3^, ^4^ The present review critically examines the available evidence linking glucocorticoid excess in CS to telomere dynamics and epigenetic remodeling, evaluates why this ostensibly ideal natural experiment has not yet yielded definitive answers, and proposes a research agenda to resolve the outstanding questions.

## 2 TELOMERE BIOLOGY UNDER GLUCOCORTICOID INFLUENCE

Telomeres are protective nucleoprotein structures capping ends of linear chromosomes and defending the integrity of the genome. In humans, telomeres span several kilobase pairs and are constituted by thousands of repeats of the hexanucleotide TTAGGG.^20^ They are composed of a non‐coding, double‐stranded for most of its length with a short single‐stranded overhang at the 3′‐OH end and associated proteins that form the shelterin complex. These complexes formed by six telomere‐specific proteins associate with this sequence and protect chromosome ends.^20^, ^21^ Telomeres shorten with every cell division due to the inability of DNA polymerases to replicate the ends of linear DNA. To work around this problem, telomeres are elongated by the telomerase ribonucleoprotein complex which comprises the telomerase reverse transcriptase (TERT), its RNA component (TERC), and other regulatory proteins such as dyskerin (DKC1).^22^ There are other ways to maintain telomere length, such as alternative lengthening of telomeres (ALT), which relies on homologous recombination between telomeric sequences.^23^

Progressively telomeres shorten with every cell division in most adult cells, including stem cells, which eventually leads to cellular senescence, associated pathologies, and it is factor limiting for human lifespan.^20^, ^24^ Telomere length shortens with aging and shorter telomeres seem to be linked with age‐related disease as well as diabetes mellitus type 2, cardiovascular disease, inflammatory states and poor lipid profile and high blood pressure.^25^, ^26^, ^27^ Therefore, if telomere length is not repaired and reaches a critical length (ca 400–1000 nucleotides), cells can either die or stay in a postmitotic state. Recent advances in the understanding of telomere biology have clarified that short telomeres are predictive of greater disease burden and mortality.^28^ Furthermore, they are one important biomarker of the aging process and in some diseases of human aging‐related processes.^29^, ^30^ Conversely, the majority cancer cells have elevated telomerase levels or activated ALT mechanisms, reaching proliferative immortality with maintenance of telomere lengths.^31^, ^32^

While the underlying mechanisms are unclear, a variety of factors such as epigenetic and environmental effects can influence and modulate the process of telomere attrition (telomere erosion or shortening). In this context, oxidative stress is thought to be the primary cause of telomere shortening in vertebrates.^20^, ^33^ Oxidative stress is a phenomenon caused by an imbalance between the production and accumulation of oxygen reactive species (ROS) in cells and tissues and the ability of a biological system to regulate the cell oxidative balance.^34^, ^35^ Oxidative damage occurs by ROS, such as free radicals, which are mainly produced by immune cells and metabolic processes.^36^, ^37^ The excess ROS can cause damage to various biomolecules, such as proteins, lipids, and DNA, and importantly, telomeres.^38^, ^39^, ^40^ Because telomeric repeats are high in guanine content, they are particularly prone to oxidative damage, which leads to telomere shortening.^13^, ^41^, ^42^, ^43^

The central neuro‐endocrine stress response system, the hypothalamic–pituitary–adrenal (HPA) axis, is answerable for stimulating a state of physiological arousal in response to stressful stimuli through a cascade of actions leading to glucocorticoid release.^14^, ^44^ The association between telomere dynamics and oxidative stress may also be mediated by glucocorticoids (GCs). Under normal conditions, upregulation of stress mediators including noradrenaline and GCs is helpful and confers adaptability to stressors, keeping levels within acceptable dynamic ranges. Glucocorticoids may affect the oxidative balance through either genomic, increasing the expression of antioxidant genes,^45^ or non‐genomic mechanisms (modulation of telomerase activity).^46^, ^47^ On the other hand, if activation of stress systems becomes repeated or excessive, with prolonged exposure to high GCs, constant activity can result in cellular stress and reduction of antioxidant defenses.^47^, ^48^

One key direct pathway is via the effect of glucocorticoids on telomere maintenance. Glucocorticoids can modulate telomere biology through both transcriptional regulation of telomerase components and indirect effects on cellular stress pathways that influence telomere stability.^49^, ^50^ In primary human T lymphocytes, exposure to cortisol has been shown to produce a marked reduction in telomerase activity accompanied by decreased transcription of the catalytic subunit hTERT, an effect observed in both CD4+ and CD8+ subsets and during primary and secondary stimulation.^47^ Mechanistically, glucocorticoid receptor (GR) activation can alter transcriptional programs via glucocorticoid response elements (GREs) and cross‐talk with other transcription factors that regulate hTERT expression, thereby providing a direct molecular route by which systemic cortisol elevations might suppress telomerase capacity.^49^, ^50^

Despite the mechanistic plausibility of cortisol‐driven telomerase suppression established in lymphocyte cell culture models, direct measurement of telomerase activity in cells derived from CS patients has not been reported in the published literature. The available evidence rests exclusively on in vitro studies demonstrating that exogenous cortisol reduces telomerase activity and hTERT transcription in isolated T lymphocytes,^47^ and on the indirect inference that this mechanism operates in vivo to produce the telomere shortening observed in some CS cohorts. This evidential gap is consequential: telomerase activity is dynamically regulated and highly context‐dependent, varying with cell activation state, proliferative history, and local cytokine environment, all of which are profoundly altered in CS. It is therefore possible that glucocorticoid‐driven lymphopenia, altered T‐cell activation thresholds, and chronic inflammation modify the net telomerase activity in CS patients in ways that cannot be predicted from in vitro models alone. Direct quantification of telomerase activity, using the TRAP (Telomeric Repeat Amplification Protocol) assay in sorted lymphocyte subpopulations from CS patients at diagnosis, during active disease, and after remission,^51^ would provide the first in vivo human evidence linking hypercortisolism to telomerase suppression and would substantially strengthen the mechanistic foundation of the dual‐pathway model proposed in this review. The absence of such data currently represents one of the most direct and addressable gaps in the CS‐aging literature.

Despite this mechanistic plausibility, translation from telomerase suppression to measurable telomere shortening in vivo is not straightforward.^18^, ^52^ Telomerase inhibition may be transient or cell‐type restricted, and detectable shortening at the organismal level requires sufficient cell turnover and cumulative replication stress; accordingly, short‐term or cell‐context‐limited reductions in telomerase do not invariably result in immediate telomere attrition.^18^, ^50^ Experimental studies in cultured human fibroblasts exposed chronically to physiological concentrations of cortisol or dexamethasone failed to demonstrate accelerated telomere shortening over multiple passages, suggesting that cell proliferation rate, lineage identity and metabolic context determine whether glucocorticoid‐induced telomerase changes translate to net length loss.^18^

Conversely, longitudinal human and animal studies provide evidence that elevated glucocorticoid exposure associates with accelerated telomere attrition under ecological or clinical conditions characterized by chronic stress.^53^, ^54^ For example, larger cortisol responses to acute psychosocial stress forecast greater leukocyte telomere shortening over multi‐year follow‐up in adults, and wild mammal models with sustained high glucocorticoid levels show faster telomere erosion, linking systemic HPA activation to cumulative telomeric damage in vivo.^53^, ^54^ Clinical studies in Cushing's syndrome (CS) show mixed: in a cohort of 77 CS patients versus matched controls, leukocyte telomere length measured by TRF (telomere restriction fragment) did not differ cross‐sectionally, but in a longitudinal subset, telomere length was significantly shorter during active disease than after remission of hypercortisolism.^55^ In pediatric CS, telomere length was not significantly shorter during active disease compared to controls, but after treatment remission, a detectable shortening relative to controls was observed; metabolic markers such as triglycerides and blood pressure correlated with telomere length changes.^56^ Moreover, associations have been reported between telomere length shortening in CS and comorbid conditions (e.g., dyslipidemia, inflammation) rather than directly with measured cortisol levels or duration of hypercortisolism.^57^ Conversely, in a cross‐species study including human CS patients, cured CS patients, and healthy controls, telomere length was significantly reduced (~40.8%) in active CS relative to controls, with partial recovery after remission; telomere length also correlated with duration of hypercortisolism.^58^ To facilitate interpretation of the heterogeneous clinical evidence, Table 1 summarizes key studies examining telomere length (TL) in the context of Cushing's syndrome and glucocorticoid exposure, with emphasis on methodological differences that may account for discrepant findings.

**TABLE 1:** Telomere length studies in Cushing's syndrome.

Author, year	Assay	Tissue/cell type	*N* (CS/controls)	Design	Comorbidities considered	CS subtype/disease duration	Treatment status	Key TL findings	Limitations
Aulinas et al.^55^	TRF–Southern blot	Total white blood cells (whole blood)	77 CS/77 matched controls	Mixed: cross‐sectional + longitudinal subset (*n* = 15)	Age, BMI, lipid profile, adrenal function, glucose	59 pituitary, 17 adrenal, 1 ectopic; 21 active disease	Mixed (active and remission)	No significant cross‐sectional TL difference; longitudinally, TL shorter during active disease versus remission (*p* < .05); no correlation with cortisol levels or disease duration	Small longitudinal subset (*n* = 15, all women); TRF measures mean TL without cell‐type resolution; no correction for lymphocyte‐to‐neutrophil ratio
Aulinas et al.^57^	TRF–Southern blot	Total white blood cells (whole blood)	77 CS/77 controls (same cohort as above)	Cross‐sectional	Dyslipidemia, hypertension, obesity, T2DM; IL‐6, CRP, adiponectin (subgroup *n* = 32)	59 pituitary, 17 adrenal, 1 ectopic; 21 active disease	Mixed (active and remission)	TL shorter in dyslipidemic CS patients (7328 vs. 7957 bp, *p* < .05); dyslipidemia and IL‐6/CRP were main predictors of TL; disease activity and cortisol not independent predictors	Same cohort as 2014 study; no cell sorting; inflammatory markers available in only 32/77 patients; confounding by metabolic factors not fully disentangled
Tatsi et al.^56^	Flow‐FISH	Peripheral lymphocyte subpopulations (total, naive T‐cells, and NK cells)	10 pediatric CD/age‐matched controls	Longitudinal (active disease + ~1 year post‐treatment)	Triglycerides, blood pressure, total cholesterol	Pituitary (Cushing disease only); mean age 13.3 years	Pituitary surgery (cure confirmed)	Total lymphocyte TL not shorter during active disease (*p* = .13); shorter at post‐treatment follow‐up (*p* = .031); naive T‐cell TL ~14.5% shorter in active disease (*p* = .004); TL correlated with triglycerides and blood pressure	Very small sample (*n* = 10); pilot study; lymphocytes susceptible to GC‐induced lymphopenia; post‐treatment shortening may reflect rebound cell turnover after lymphopenia resolution
Lee et al.^58^	qPCR (relative T/S ratio)	Leukocytes (whole blood gDNA) + cross‐species (rat, mouse, and cell line)	32 CS (9 active/23 cured)/32 controls	Cross‐sectional (human CS) + cross‐species	Age, sex; limited metabolic data reported	Predominantly pituitary; varied duration	Varied (mostly post‐surgery)	TL ~40.8% shorter in active CS vs. controls (*p* = .006); cured patients showed partial recovery; TL correlated with duration of hypercortisolism (*p* = .007); association confirmed across species	Very small active CS group (*n* = 9); qPCR has higher assay variance than TRF; no cell‐type correction; discrepant from Aulinas^55^ likely due to different assay sensitivity
Athanasoulia‐Kaspar et al.^59^	qPCR	Leukocytes (whole blood)	115 NFPA/106 matched controls	Cross‐sectional	GH deficiency, IGF‐1, glucocorticoid substitution dose, metabolic parameters	Non‐functioning pituitary adenoma (NFPA); exogenous GC via substitution therapy	Post‐treatment (substitution therapy)	Shorter TL in NFPA patients versus controls; TL inversely correlated with cumulative glucocorticoid substitution dose; GH deficiency not independently associated with TL	NFPA population—exogenous GC exposure, not endogenous hypercortisolism; qPCR methodology; cross‐sectional only; no CS subtype stratification

*Note*: Summary of clinical studies examining telomere length in Cushing's syndrome and glucocorticoid exposure. To facilitate interpretation of the heterogeneous clinical evidence, key studies are compared with emphasis on methodological differences that may account for discrepant findings.

Abbreviations: BMI, body mass index; CD, Cushing's disease; CRP, C‐reactive protein; CS, Cushing's syndrome; Flow‐FISH, flow cytometry–fluorescence in situ hybridization; GC, glucocorticoid; GH, growth hormone; IL‐6, interleukin‐6; NFPA, non‐functioning pituitary adenoma; NK, natural killer; qPCR, quantitative polymerase chain reaction; TL, telomere length; TRF, terminal restriction fragment; T2DM, type 2 diabetes mellitus.

The discrepant findings summarized in Table 1 are not readily attributable to biological variability alone, but rather reflect systematic methodological differences that must be critically considered when interpreting the available evidence. The most salient source of inconsistency is assay choice: Aulinas et al.^55^, ^57^ employed TRF–Southern blot, widely regarded as the reference standard for absolute TL measurement, whereas Lee et al. and Athanasoulia‐Kaspar et al.^58^, ^59^ used quantitative PCR, which yields relative T/S ratios subject to considerably greater amplification variance. Comparative platform studies have reported only modest correlations between these two methods (*R* ^2^ ≈ 0.27), with qPCR typically showing higher between‐study variability.^60^ This methodological divergence alone may explain why Lee et al.^58^ detected a ~40.8% TL reduction in active CS while Aulinas et al.^55^ found no cross‐sectional difference using the same patient population design but a more precise assay.

A second critical factor is cell‐type specificity. All studies except Tatsi et al.^56^ measured TL in unsorted mixed leukocytes or whole blood. This is particularly consequential in CS, where chronic glucocorticoid excess induces a well‐documented lymphopenia and shifts the leukocyte composition toward neutrophil predominance.^47^ Because lymphocytes carry substantially longer telomeres than neutrophils, any GC‐driven reduction in the lymphocyte‐to‐neutrophil ratio will artifactually lower bulk leukocyte TL independently of true telomeric attrition at the cellular level.^60^, ^61^ This confound has not been corrected in any of the adult CS studies reviewed here, representing a fundamental limitation in the interpretation of their TL data.

Third, sample size constraints undermine the reliability of effect size estimates across all available studies. The active CS group in Lee et al.^58^ comprised only nine patients, making that study statistically underpowered to detect modest TL differences with precision. Similarly, the longitudinal subset in Aulinas et al.^55^ included only 15 women, precluding generalization. Fourth, study design introduces temporal bias: cross‐sectional measurements capture cumulative lifetime TL exposures that predate the diagnosis of CS, whereas longitudinal follow‐up, as partially performed by Aulinas^55^ and Tatsi,^56^ can begin to disentangle disease‐driven from age‐driven attrition. Finally, heterogeneity in comorbidity adjustment is a pervasive issue: Aulinas et al.^57^ demonstrated that dyslipidemia and systemic inflammation, rather than hypercortisolism per se, were the primary predictors of TL shortening in CS, underscoring that metabolic confounders must be explicitly modeled in future studies.^57^ Taken together, these considerations suggest that apparent null results in CS telomere studies may reflect measurement insensitivity or cellular composition confounding rather than a true biological absence of GC‐driven telomere attrition.

### 2.1 Glucocorticoid‐induced aging (non‐telomeric mechanisms)

Although telomere attrition is a widely used marker of cellular aging, it represents only one component of a complex and multifactorial aging process. Chronic glucocorticoid exposure has been shown to influence several telomere‐independent aging pathways, including oxidative stress, mitochondrial dysfunction, and inflammation‐driven cellular senescence. For example, sustained glucocorticoid signaling markedly increases mitochondrial reactive oxygen species (ROS) and oxidative damage.^16^ In cultured cells, high‐dose dexamethasone upregulated mitochondrial fission factor Drp1 and accelerated oxidative phosphorylation, producing excess superoxide.^62^ In line with this, glucocorticoids induce mitochondrial fragmentation and depolarization: animal studies show that chronic corticosterone damages mitochondrial ultrastructure (in hippocampus, cortex and other regions) and reduces respiratory‐chain activity.^16^, ^63^ The resulting dysfunctional mitochondria generate more ROS and elicit pro‐apoptotic signaling. Moreover, glucocorticoids impair mitochondrial quality control: they suppress mitophagy via downregulation of BNIP3L/NIX, causing accumulation of defective mitochondria. Without effective turnover, damaged organelles persist and amplify oxidative stress.^17^, ^62^

In parallel, glucocorticoids exert complex and context‐dependent effects on autophagy and cellular proteostasis. Mechanistically, synthetic glucocorticoids such as dexamethasone can induce autophagy in multiple cell types, including chondrocytes and skeletal muscle, largely through modulation of the PI3K/AKT/mTOR signaling axis.^17^, ^64^ In chondrocytes, dexamethasone suppresses AKT and mTOR activity, leading to increased autophagic activity that may initially serve a cytoprotective role by mitigating oxidative stress and maintaining cellular homeostasis.^65^, ^66^ Similarly, in skeletal muscle, dexamethasone activates autophagy and mitophagy programs, contributing to organelle turnover and quality control; however, this response is tightly coupled to the activation of catabolic pathways and the progression of muscle atrophy.^67^

Importantly, accumulating evidence indicates that glucocorticoid‐mediated regulation of autophagy is not uniformly stimulatory. In certain contexts, such as immune cells or infection models, glucocorticoids suppress autophagic flux through enhancement of mTOR signaling and downregulation of core autophagy‐related genes, thereby impairing autophagosome maturation and degradative capacity.^68^ These findings support a model in which glucocorticoids induce an early or compensatory activation of autophagy, followed by a dysregulated or insufficient autophagic flux under chronic exposure. This imbalance, together with increased proteolysis via the ubiquitin–proteasome system and suppression of anabolic signaling pathways, contributes to disrupted proteostasis, accumulation of damaged proteins and organelles, and progressive cellular dysfunction.^69^

Chronic glucocorticoid excess also provokes a pro‐inflammatory milieu. Although glucocorticoids are acutely anti‐inflammatory, prolonged hypercortisolism paradoxically maintains low‐grade systemic inflammation. Patients with active Cushing's disease have elevated circulating cytokines (IL‐6, IL‐1β, and TNF‐α) compared to controls, and these remain high long after cure.^70^ Inflammatory cytokines can induce cellular senescence. For example, exogenous IL‐6 (together with soluble IL‐6Rα) triggered premature senescence in human fibroblasts,^71^ and SASP factors like IL‐6/IL‐8 are known to reinforce senescence in neighboring cells.^71^, ^72^ Thus, sustained glucocorticoid excess drives a feed‐forward loop: oxidant injury and mitochondrial decay provoke senescence, while chronic inflammation and cytokine release (driven by tissue damage and dysregulated immunity) induce further paracrine senescence.

**FIGURE 2:**
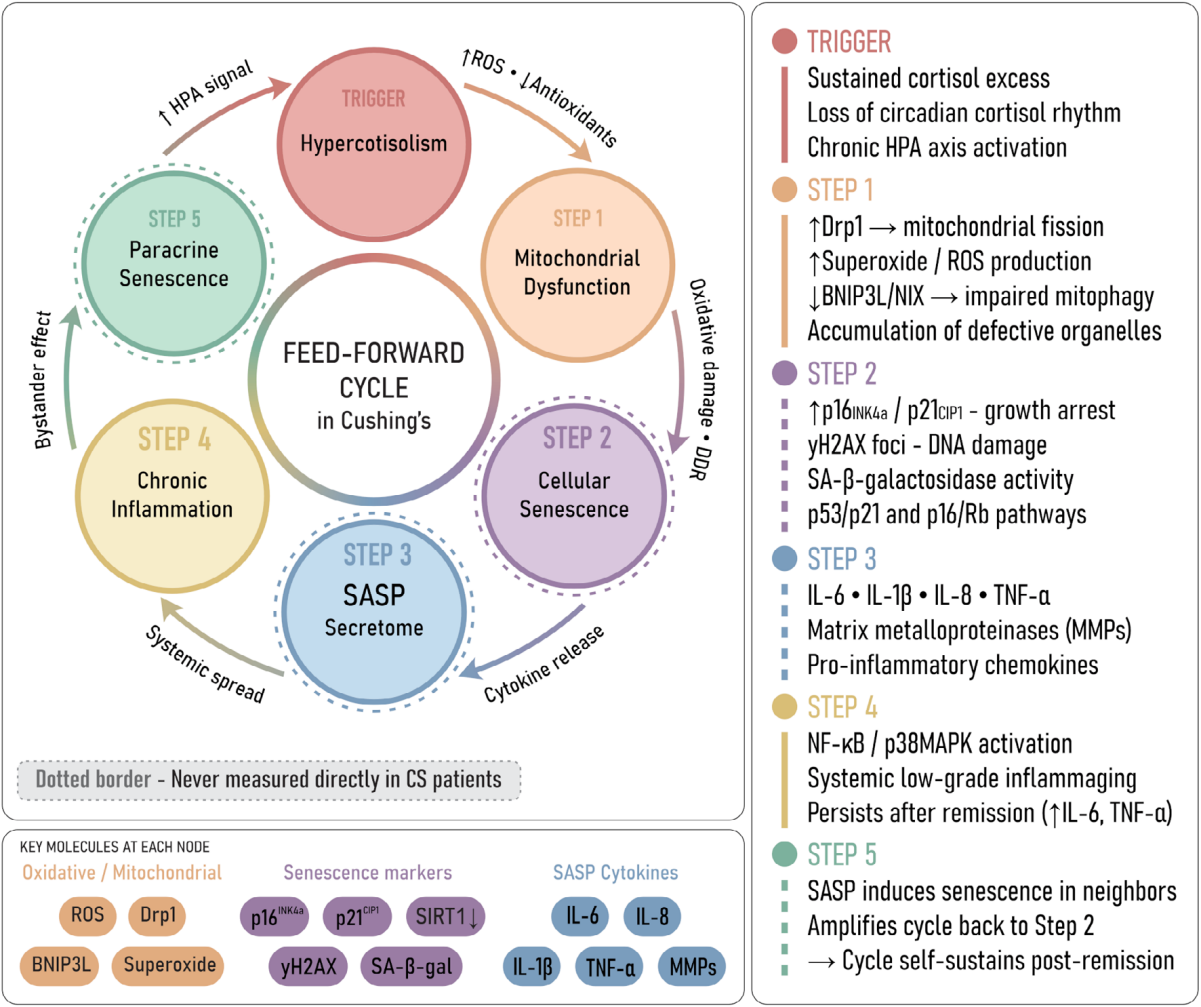
The SASP feed‐forward cycle in Cushing's syndrome. Chronic endogenous hypercortisolism initiates a self‐amplifying senescence cascade. Sustained glucocorticoid excess drives mitochondrial fragmentation (↑Drp1), impairs mitophagy (↓ BNIP3L/NIX), and generates excess reactive oxygen species (ROS)—collectively promoting cellular senescence characterized by p16^INK4a^/p21^CIP1^ upregulation and SA‐β‐galactosidase activity (Step 2). Senescent cells release a pro‐inflammatory secretome (SASP: IL‐6, IL‐1β, IL‐8, TNF‐α, matrix metalloproteinases; Step 3), sustaining low‐grade systemic inflammation via NF‐κB/p38MAPK activation (Step 4). SASP factors induce paracrine senescence in neighboring cells (Step 5), which re‐enters and amplifies the cycle back to Step 2. Critically, nodes marked with dotted borders (Steps 2, 3, and 5) represent processes inferred from experimental models that have never been directly measured in CS patients—constituting a high‐priority gap for future research. BNIP3L/NIX, BCL2‐interacting protein 3‐like; CS, Cushing's syndrome; DDR, DNA damage response; Drp1, dynamin‐related protein 1; GC, glucocorticoid; HPA, hypothalamic–pituitary–adrenal; IL, interleukin; MMP, matrix metalloproteinase; NF‐κB, nuclear factor kappa BROS, reactive oxygen species; SA‐β‐gal, senescence‐associated β‐galactosidase; SASP, senescence‐associated secretory phenotype; TNF‐α, tumor necrosis factor alpha.

These telomere‐independent pathways, mitochondrial dysfunction, oxidative stress, disrupted autophagy/mitophagy, and cytokine‐mediated senescence, together recapitulate features of accelerated aging. Importantly, they do not rely on telomere attrition per se, but converge on common aging hallmarks (reactive oxygen damage, DNA damage response, NF‐κB activation, p53/p21 and p16/Rb pathways).^16^, ^17^ Indeed, chronic glucocorticoid models show elevated p21 and p16 levels and reduced sirtuin‐1 activity, consistent with premature growth arrest.^73^ In sum, a breadth of human, animal and cell studies indicate that prolonged cortisol/CORT exposure drives multifactorial cellular wear and tear, producing an “aged” phenotype even without direct telomere shortening.^16^, ^71^ The proposed feed‐forward cycle linking glucocorticoid‐driven mitochondrial dysfunction to cellular senescence and SASP propagation is illustrated in Figure 2.

Despite the mechanistic plausibility of glucocorticoid‐driven cellular senescence outlined above, direct markers of senescence have rarely been quantified in patients with CS. The canonical markers of cellular senescence include upregulation of cyclin‐dependent kinase inhibitors p16^INK4a^ (encoded by *CDKN2A*) and p21^CIP1^ (encoded by *CDKN1A*), activation of the DNA damage response (γH2AX foci), and senescence‐associated β‐galactosidase (SA‐β‐gal) activity, collectively representing the most direct cellular readouts of growth arrest and senescence induction.^74^, ^75^ While chronic glucocorticoid exposure has been shown to elevate p21 and p16 levels and reduce sirtuin‐1 activity in experimental models, consistent with premature growth arrest, equivalent measurements in circulating or tissue‐derived cells from CS patients are virtually absent from the published literature. This represents a fundamental evidential gap: the entire mechanistic argument linking hypercortisolism to accelerated cellular aging in CS rests on indirect biomarkers (telomere length, DNA methylation age) and experimental models, without confirmation that bona fide senescent cells accumulate in CS patients at rates exceeding those of age‐matched controls. Future studies should incorporate flow cytometric quantification of p16^INK4a^ and p21^CIP1^ expressing leukocyte subpopulations, as well as SA‐β‐gal activity in accessible cell types, alongside the molecular aging biomarkers discussed in this review.^76^ Such measurements would provide the most direct available evidence for or against genuine glucocorticoid‐driven premature cellular senescence in humans.

Closely related to the question of senescence marker quantification is the characterization of the senescence‐associated secretory phenotype (SASP) in CS. The SASP comprises a pro‐inflammatory secretome, including IL‐6, IL‐1β, TNF‐α, IL‐8, and matrix metalloproteinases, released by senescent cells that reinforces local and systemic inflammation and induces paracrine senescence in neighboring tissues.^77^, ^78^ Critically, the cytokine profile documented in active Cushing's disease, persistently elevated circulating IL‐6, IL‐1β, and TNF‐α that remain high long after biochemical cure, is strikingly congruent with canonical SASP composition. However, no published study has formally characterized whether this inflammatory profile in CS originates from senescent cells exhibiting SASP, or whether it reflects glucocorticoid‐driven immune dysregulation through independent mechanisms such as NF‐κB activation and impaired anti‐inflammatory feedback. This distinction carries significant mechanistic and therapeutic implications: notably, in vitro evidence demonstrates that cortisol and corticosterone can paradoxically suppress selected SASP components via glucocorticoid receptor‐mediated inhibition of IL‐1α signaling and NF‐κB transactivation.^79^ If the chronic inflammation of CS is genuinely SASP‐driven, it would suggest that senolytic or senomorphic interventions targeting the SASP could complement standard endocrine treatment in reducing the long‐term comorbidity burden of CS, even after successful cortisol normalization.^80^ Conversely, if the inflammatory profile reflects direct glucocorticoid immunomodulation, it would be expected to resolve more completely with remission. Prospective studies combining SASP profiling with senescence marker quantification before and after treatment represent a high‐priority research direction that could fundamentally reframe the therapeutic approach to CS‐associated morbidity.

### 2.2 Endocrine and circadian drivers of aging in Cushing's syndrome

Beyond sustained hypercortisolism, Cushing's syndrome is characterized by marked disruption of the normal circadian rhythm of cortisol secretion, a pattern that is essential for metabolic homeostasis, immune regulation, and cellular repair processes. A signature feature is loss of the normal circadian cortisol rhythm: instead of a high morning peak and low night level, cortisol remains inappropriately elevated throughout the day. This blunted diurnal curve resembles the pattern seen in older adults (age‐related flattening of cortisol rhythm) and has profound effects on physiology.^81^ Recent data show that active Cushing's flattens peripheral clock gene oscillations. For example, PBMCs from patients with endogenous Cushing's have markedly damped expression of core clock genes (*PER1‐3*, *CLOCK*, and *TIMELESS*), reflecting a loss of circadian immune cycling.^82^ Such chronodisruption promotes “inflammaging”: in mouse models, chronic circadian misalignment shortens lifespan (hazard ratio ~ 20×) and upregulates pro‐inflammatory pathways in peripheral tissues.^83^ By analogy, the constant hypercortisolism in Cushing's likely induces a similar state of systemic inflammation and impaired cellular repair.

Other endocrine axes are similarly affected. Hypercortisolism suppresses growth hormone (GH) secretion: most adults with active Cushing's exhibit GH deficiency as a direct consequence of cortisol excess.^84^ GH/IGF‐1 signaling normally promotes muscle and bone anabolism and metabolic homeostasis; its attenuation leads to aging phenotypes. Untreated GH deficiency (as often seen in Cushing's) causes increased visceral adiposity, muscle wasting, osteoporosis, dyslipidemia, and insulin resistance, essentially the features of somatopause and metabolic syndrome. Importantly, restoring GH (or removing the cortisol excess) partially reverses these effects: GH therapy in Cushing's patients increases lean mass, reduces fat, and normalizes lipid profiles, indicating that cortisol‐induced somatopause drives accelerated tissue aging. Paradoxically, IGF‐1 levels in Cushing's may be high during active disease (possibly due to cortisol‐induced hepatic IGF‐1 production), but the physiological GH pulse pattern and peripheral IGF‐1 signaling remain disrupted.^84^, ^85^

Metabolic endocrine aging may also occur in Cushing's syndrome. Cortisol antagonizes insulin action: patients with Cushing's uniformly exhibit severe insulin resistance and hyperglycemia. This glucocorticoid‐driven insulin resistance is post‐receptor and often improves after cure.^86^ Chronic hyperglycemia itself is pro‐oxidant and pro‐senescent, causing glycation and activating inflammatory pathways (advanced glycation end products, NF‐κB). Thus Cushing's‐associated diabetes accelerates vascular and cellular aging. The stress‐induced catecholaminergic axis is also involved: cortisol increases adrenergic receptor sensitivity, and stress‐level epinephrine further inhibits autophagy, compounding organelle damage.^17^ Collectively, the endocrine milieu of Cushing's mimics multiple hallmarks of aging.

Finally, circadian and endocrine dysregulation in Cushing's converge on core aging pathways. Loss of cortisol rhythm and chronic inflammation enhance NF‐κB/p38MAPK signaling and SASP expression, driving cells into senescence. Impaired GH/IGF‐1 and insulin signaling alter FOXO and mTOR pathways, promoting catabolism and reducing stress resistance. In experimental models, chronic glucocorticoid exposure alone induces DNA damage and cellular senescence in various tissues. The clinical comorbidities of Cushing's (osteoporosis, sarcopenia, diabetes, and atherosclerosis) illustrate this accelerated aging.^82^, ^83^ Collectively, these endocrine and circadian disruptions converge on the same molecular aging hallmarks as glucocorticoid‐driven telomere attrition, NF‐κB activation, SASP expression, impaired proteostasis, and mitochondrial dysfunction, reinforcing the view that CS represents a multi‐axis progeroid state rather than a single‐pathway aging model.

The telomeric and non‐telomeric aging pathways discussed above are not, however, independent of the epigenetic landscape. Glucocorticoid excess simultaneously remodels DNA methylation patterns in ways that may amplify both telomere instability and the broader cellular senescence program. The following section examines this epigenetic dimension, from the most replicated locus‐specific findings to genome‐wide remodeling and its potential to accelerate biological age as captured by methylation‐based clocks.

## 3 EPIGENETIC MODULATION: DNA METHYLATION, GLUCOCORTICOID REMODELING, AND BIOLOGICAL AGING IN CUSHING'S SYNDROME

### 3.1 DNA methylation as an epigenetic mechanism

**FIGURE 3:**
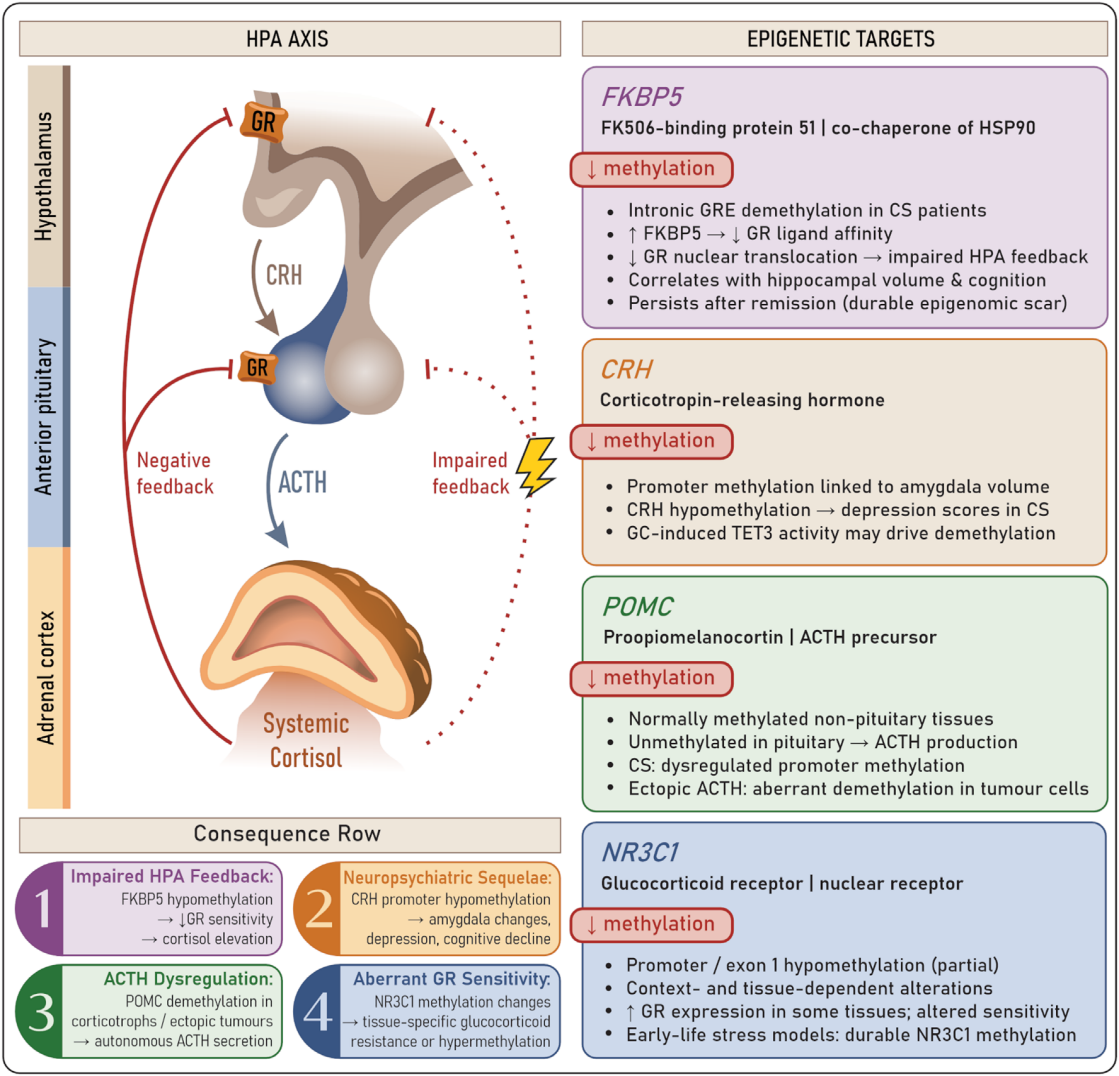
The hypothalamic–pituitary–adrenal (HPA) axis and its epigenetic targets in Cushing's syndrome. Under normal physiology, cortisol exerts negative feedback on both the hypothalamus and anterior pituitary via the glucocorticoid receptor (GR), limiting further CRH and ACTH release. In Cushing's syndrome, this feedback is impaired (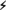). Chronic cortisol excess acts through GR to induce TET3‐mediated active DNA demethylation, converting 5‐methylcytosine (5mC) to 5‐hydroxymethylcytosine (5‐hmC) at glucocorticoid response elements (GREs), producing durable hypomethylation at four key loci. *FKBP5* intronic CpG hypomethylation amplifies GR insensitivity and correlates with hippocampal volume loss and cognitive outcomes in CS patients. *NR3C1* promoter methylation undergoes context‐dependent alterations that shift GR expression and sensitivity across tissues. POMC is normally silenced by promoter methylation in non‐pituitary tissue but becomes aberrantly demethylated in corticotroph adenomas and ectopic tumors, driving autonomous ACTH production. CRH promoter hypomethylation has been linked to amygdala structural changes and depression scores. ACTH, adrenocorticotropic hormone; CRH, corticotropin‐releasing hormone; CS, Cushing's syndrome; FKBP5, FK506‐binding protein 51; GR, glucocorticoid receptor; GRE, glucocorticoid response element; HPA, hypothalamic–pituitary–adrenal; NR3C1, nuclear receptor subfamily 3 group C member 1; POMC, proopiomelanocortin; TET3, ten‐eleven translocation methylcytosine dioxygenase 3; 5mC, 5‐methylcytosine; 5‐hmC, 5‐hydroxymethylcytosine.

Epigenetic regulation shapes gene expression without altering the underlying DNA sequence, and three principal mechanisms have been described: histone modification, non‐coding RNA species, and DNA methylation.^87^ DNA methylation involves the transfer of a methyl group to the fifth carbon of cytosine residues, predominantly at CpG dinucleotides, to form 5‐methylcytosine (5mC), a reaction catalyzed by a family of DNA methyltransferases (DNMTs).^88^, ^89^ The canonical DNMTs include DNMT1, which maintains methylation patterns during DNA replication, and DNMT3A and DNMT3B, which establish de novo methylation on unmodified DNA.^90^, ^91^ CpG sites cluster into CpG islands associated with gene promoters, which are typically unmethylated in actively transcribed genes; aberrant hypermethylation of these islands is generally linked to transcriptional silencing and has been implicated in a broad range of diseases.^92^, ^93^, ^94^ Beyond its role in transcriptional regulation, DNA methylation participates in genomic imprinting, X‐chromosome inactivation, silencing of repetitive elements, and preservation of chromosomal stability.^95^, ^96^ Importantly, a related modification (5‐hydroxymethylcytosine (5‐hmC)) is generated by TET enzyme‐mediated oxidation of 5mC and represents an intermediate in active DNA demethylation, adding a further layer of dynamic epigenetic regulation relevant to glucocorticoid biology, as discussed below. The principal epigenetic targets of chronic glucocorticoid excess within the HPA axis—FKBP5, NR3C1, POMC, and CRH—and their methylation status in CS are illustrated in Figure 3.

### 3.2 Glucocorticoid‐driven methylation changes in CS: Established findings

#### 3.2.1 *FKBP5* hypomethylation—the most replicated glucocorticoid‐epigenetic signal

The most consistently replicated epigenetic consequence of glucocorticoid excess is hypomethylation of the *FKBP5* gene. *FKBP5* (FK506‐binding protein 51) encodes a co‐chaperone of the HSP90 complex that modulates glucocorticoid receptor (GR) sensitivity by decreasing ligand affinity and delaying GR nuclear translocation, thereby acting as a rapid feedback regulator of HPA‐axis signaling.^97^ Glucocorticoid‐responsive demethylation at intronic GREs amplifies *FKBP5* induction after stress, producing long‐lasting increases in *FKBP5* expression and altered HPA feedback.^15^ In patients with endogenous CS, targeted analyses confirm reduced intronic methylation of *FKBP5* relative to healthy controls, and specific CpG hypomethylation within *FKBP5* has been correlated with hippocampal volume loss and cognitive outcomes.^8^ Experimental studies further demonstrate that acute glucocorticoid challenge induces rapid, locus‐specific methylation changes across *FKBP5* regulatory regions in blood, mirroring epigenetic responses observed in brain tissue in model systems.^98^ These findings establish *FKBP5* hypomethylation as a durable, clinically meaningful epigenetic scar of chronic hypercortisolism in CS.

#### 3.2.2 Subtelomeric methylation and its link to telomere biology

Beyond gene‐specific effects, subtelomeric DNA methylation has been implicated in telomere stability and length regulation. Human studies report correlations between subtelomeric methylation and telomere length, and disorders of DNMT function, such as DNMT3B‐related ICF syndrome, exhibit subtelomeric hypomethylation together with telomeric instability, directly linking methylation state to telomere biology.^19^ This provides a mechanistic bridge between the epigenetic remodeling induced by chronic glucocorticoid excess and the telomere attrition discussed in Section 2: *FKBP5* hypomethylation and broader methylome dysregulation could indirectly destabilize telomeres via altered chromatin states, dysregulated DNA damage responses, and shifts in oxidative stress handling, thereby coupling HPA dysregulation to cellular senescence.^15^

### 3.3 Beyond *FKBP5* : Broader epigenomic remodeling by glucocorticoids

While *FKBP5* has emerged as the most consistently replicated glucocorticoid‐responsive locus, accumulating evidence indicates that chronic glucocorticoid exposure induces broader epigenetic remodeling extending well beyond a single gene:

- Additional GC‐target genes and pathways: Blood methylation of the CRF (corticotropin‐releasing factor) promoter has been linked to amygdala volume and depression scores in CS patients.^99^ A genome‐wide study of remitted CS identified 337 differentially methylated genes, with the most enriched gene‐ontology terms relating to retinoic acid receptor signaling, implicating neuroendocrine and developmental gene networks.^100^ Promoters of key HPA‐axis genes, including POMC and GR/NR3C1, are also GC‐responsive targets of epigenetic change.
- TET enzymes and DNA hydroxymethylation: Chronic GC exposure drives active demethylation through TET enzymes. Dexamethasone‐treated neural stem cells showed TET3‐dependent global 5mC loss and increased 5‐hmC, with Dex‐induced downregulation of DNMT3a and upregulation of TET3 required for persistent demethylation and altered gene expression. These results indicate that TET‐mediated hydroxymethylation is a key pathway by which excess cortisol reprograms the epigenome, potentially creating long‐lived hypomethylated marks that sustain gene dysregulation in CS.^101^, ^102^
- Circadian clock genes: Chronic hypercortisolism disrupts the normal circadian expression of peripheral clock genes (*CLOCK*, *BMAL1*, *PER1/2/3*, and *CRY1*) in CS patients.^82^ Even after cure, genes like *PER1* remain abnormally expressed with persistent changes in histone marks (H3K4me3, H3K27ac). GR‐induced remodeling of circadian chromatin structure, including disruption of 3D chromatin loops at the Nr1d1 locus, may underlie the metabolic and immune dysregulation observed in CS.^102^, ^103^
- Global epigenomic remodeling: Genome‐wide blood DNA methylation profiling of CS patients has identified persistent hypomethylated CpG sites linked to comorbidities including hypertension and osteoporosis. Remitted CS patients show lower overall DNA methylation than controls, with the most affected genes involving retinoic acid, thyroid hormone, and other nuclear receptor pathways.^100^, ^102^ These broad epigenomic shifts likely contribute to the persistent physiological and psychiatric features of CS that outlast biochemical remission.

### 3.4 Epigenetic clocks, DNAmTL, and mortality evidence in CS

Epigenetic clocks are statistical predictors of biological age derived from DNA methylation at selected CpG sites and have emerged as powerful biomarkers of morbidity and mortality.^104^, ^105^ Second‐generation clocks, GrimAge and GrimAge2, show superior prediction of all‐cause mortality and age‐related phenotypes compared with first‐generation tools (Horvath, Hannum),^106^, ^107^ and each 5‐year epigenetic age acceleration is associated with an appreciable increase in mortality risk across large cohorts.^104^, ^108^ Complementary to epigenetic clocks, methylation‐based telomere length estimators (DNAmTL) infer average telomere length from a CpG signature and have demonstrated stronger prediction of mortality, cancer risk, and cardiovascular outcomes than conventional TL assays in several large cohorts.^12^, ^109^, ^110^ These tools are not interchangeable, however: DNAmTL and direct TL measures correlate only modestly and can diverge at the individual level, while epigenetic clocks reflect composite exposures, including smoking, inflammation, and metabolic status, that may be partly independent of telomere dynamics.^12^, ^111^

Consistent with the elevated mortality burden of CS described in Section 1.1, these epigenetic findings carry direct clinical relevance. However, this excess mortality is multifactorial and not yet attributable solely to accelerated molecular aging. Clinical studies of TL in CS provide mixed results,^55^, ^56^, ^112^ and no published study has yet applied second‐generation epigenetic clocks to directly quantify biological age acceleration in active versus remitted CS, representing a critical evidence gap. The long‐term cortisol exposure in CS induces locus‐specific and global methylation changes that could contribute to epigenetic age acceleration,^98^, ^102^ and integrating epigenetic clocks and DNAmTL into longitudinal CS designs would allow direct interrogation of whether hypercortisolism accelerates the epigenetic clock, whether DNAmTL mediates telomere‐related risk, and how both measures relate to clinical endpoints.^113^, ^114^

The epigenetic evidence in Cushing's syndrome converges on two conclusions. First, chronic glucocorticoid excess produces durable, genome‐wide epigenetic remodeling, most robustly demonstrated at *FKBP5*, but extending to HPA‐axis genes, circadian clock *loci*, and metabolic regulatory networks that persist beyond biochemical remission and may contribute to the lasting comorbidity burden of CS.^8^, ^15^, ^100^ Second, the tools most capable of translating this epigenetic disruption into quantifiable biological aging signals, second‐generation epigenetic clocks and DNAmTL, have not yet been systematically applied to CS cohorts, leaving the central question of whether hypercortisolism genuinely accelerates epigenetic age empirically unanswered.^5^, ^106^, ^107^ Resolving this gap requires longitudinal, multi‐platform studies with explicit confounder adjustment, as outlined in Section 5.

## 4 LIMITATIONS OF AGING BIOMARKERS (TL, DNAmTL, AND EPIGENETIC CLOCKS)

**FIGURE 4:**
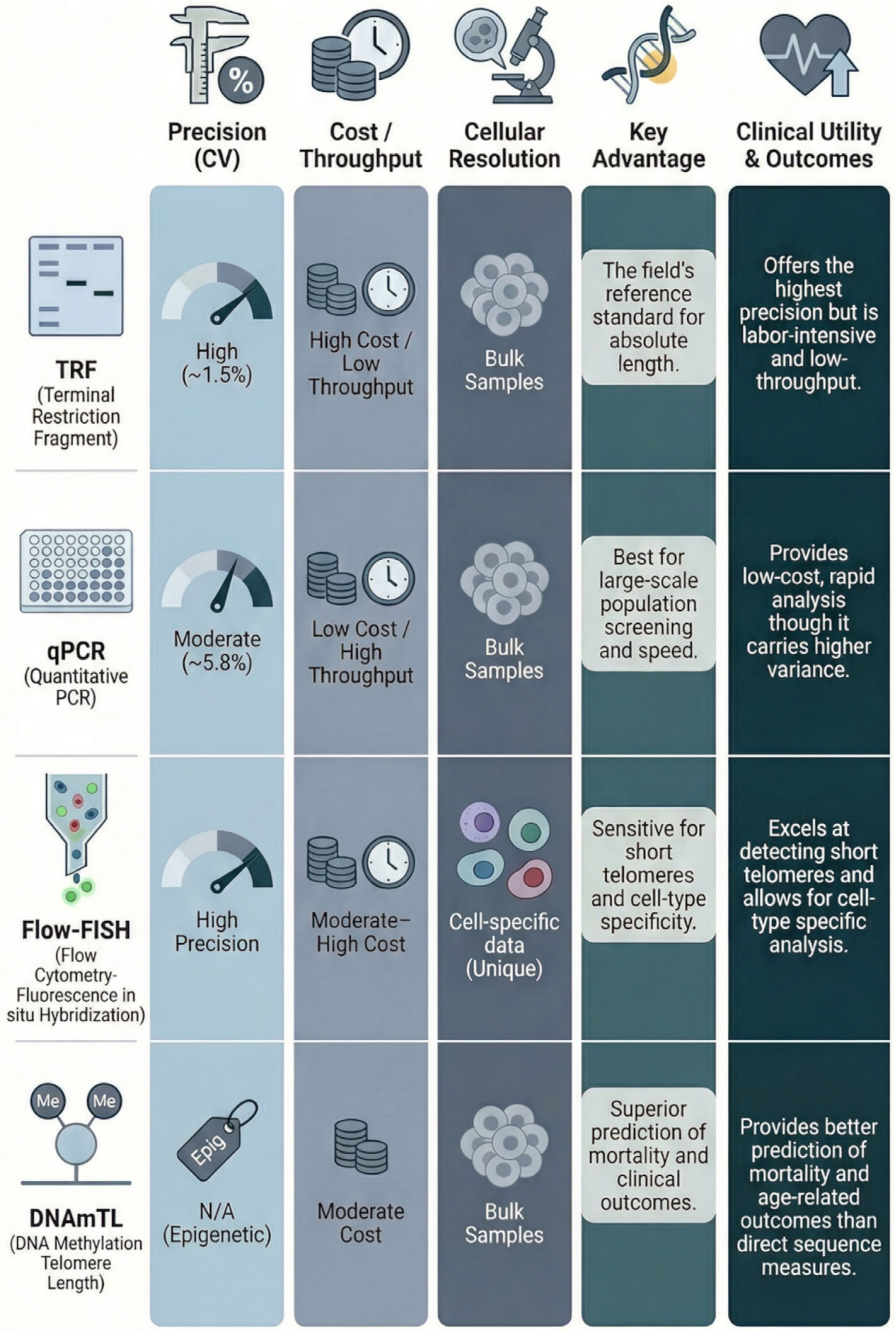
Methodological comparison of telomere length measurement platforms. While telomere attrition is a hallmark of biological aging, the choice of measurement method significantly influences data interpretation. This comparison highlights the trade‐offs between precision, cost‐efficiency, and clinical predictive power across traditional and emerging assays. Schematic comparison of the four primary assays used to assess telomere dynamics in clinical and research settings. Telomere Restriction Fragment (TRF) analysis via Southern blot remains the gold standard for absolute quantification and precision, though it requires high DNA input and is labor‐intensive. Quantitative PCR (qPCR) is the most common high‐throughput method but exhibits higher inter‐assay variance (CV ~5.8%) and limited sensitivity to the shortest telomeres. Flow‐FISH (Fluorescence In Situ Hybridization) offers superior cellular resolution by measuring telomere length in individual cell subpopulations, providing high sensitivity for the critically short telomeres that trigger senescence. DNAmTL (DNA methylation‐based telomere length) is an epigenetic estimator that reflects cellular replication history rather than physical sequence length; it has demonstrated superior prediction of mortality and can be applied across different tissue types.

Common aging biomarkers each have significant caveats. Telomere length (TL) shows large inter‐individual and technical variability. Different assays (Southern blot TRF, qPCR, and Flow‐FISH) are poorly concordant: for instance, one large study found qPCR and Southern‐blot TL measurements correlated modestly (*R* ^2^ ≈ 0.27).^115^ qPCR has higher assay variance (CV ≈ 5.8%) than Southern blot (CV ≈ 1.5%). Flow‐FISH is more precise than qPCR: flow‐FISH showed lower inter‐assay CV and greater sensitivity for short telomeres.^115^, ^116^ These differences mean that TL values (and any “biological age” derived) depend strongly on method and laboratory protocols.^116^, ^117^ A systematic comparison of these four methods and their specific limitations in the context of CS is provided in Figure 4. Pre‐analytical factors (DNA extraction, storage conditions) further perturb results.^117^ Moreover, TL varies by genetics and demographics: for example, meta‐analyses show women on average have slightly longer leukocyte TL than men,^118^ but only Southern blot assays consistently detect this sex difference. Age‐related attrition also is nonlinear: TL shortens most rapidly in early childhood, then plateaus into adulthood, complicating comparisons across ages. Critically, TL measured in blood may not reflect other organs: average TL differs by tissue (longest in testis, shortest in blood) yet correlates only moderately across tissues.^119^

The tissue‐dependence of TL is not merely a technical caveat but a fundamental biological reality with direct implications for studies in Cushing's syndrome. The largest multi‐tissue TL dataset to date, derived from over 6000 tissue samples across more than 20 tissue types from 952 donors in the GTEx project, demonstrated that while whole blood TL correlates positively with TL in most tissues, the strength of this association varies substantially across organ types, and cell division rate, local oxidative stress, and microenvironmental exposures introduce meaningful divergence between blood and metabolically relevant organs such as adipose tissue, liver, and vascular endothelium.^120^ A systematic review and meta‐analysis of cross‐tissue TL studies confirmed that tissue source exerts a significant independent effect on the magnitude of TL—health outcome associations, underscoring that blood‐based TL proxies cannot be assumed to faithfully represent the molecular aging state of pathophysiologically relevant organs.^121^ This limitation is particularly consequential in CS, where the tissues most directly implicated in excess morbidity and mortality (visceral adipose tissue, vascular endothelium, liver, and bone) are precisely those whose TL and methylation dynamics are least likely to mirror those of circulating leukocytes. Adipose tissue TL has been independently associated with adipocyte dysfunction, impaired glucose and lipid metabolism, and cardiovascular risk, with associations that are not fully recapitulated by leukocyte TL.^122^ In vascular endothelial cells, telomere‐driven senescence induces multi‐organ metabolic dysfunction, encompassing adipose tissue, brain, and skeletal muscle, through paracrine mechanisms that operate independently of blood cell telomere status.^123^

Epigenetic telomere estimators (DNAmTL) similarly have constraints. DNAmTL correlates only moderately with measured TL and with other aging clocks. In one study of children, qPCR TL and DNAmTL had poor agreement (*r* ≈ 0.20), and DNAmTL was more strongly associated with age and sex than raw TL.^110^, ^124^ Mechanistically, DNAmTL appears to reflect cellular replication history rather than telomere sequence length: in vitro, cells expressing telomerase (hTERT) maintain long telomeres but still show declining DNAmTL.^110^ Thus, DNAmTL (like measured TL) is an indirect index with its own noise and is primarily calibrated in blood leukocytes, limiting its generalizability.

DNA methylation patterns relevant to type 2 diabetes, insulin resistance, and cardiovascular disease have been identified in pancreatic islets, liver, skeletal muscle, and adipose tissue, with tissue‐specific epigenetic signatures that are not consistently reflected in parallel blood measurements.^125^ Notably, DNAmTL (unlike directly measured TL) has demonstrated transferability beyond blood: it has been validated in adipose and liver tissue and retains significant associations with cardiovascular disease and all‐cause mortality in large cohorts even when trained on non‐blood tissues, suggesting that CpG‐based methylation proxies can capture biologically relevant aging variance beyond the tissue of measurement.^110^ Nevertheless, DNAmTL remains primarily calibrated in leukocytes, and its performance in tissues with markedly different cell turnover rates, such as post‐mitotic neurons or slowly dividing hepatocytes, has not been systematically validated, limiting conclusions about tissue‐specific molecular aging in CS from blood‐based measurements alone.

First‐generation epigenetic clocks (Horvath, Hannum) are also limited by tissue and technical factors. They were trained on specific tissues and populations, so results vary by source and platform. For example, clock estimates can differ by up to ~10 years between blood and saliva from the same individual, unless using a pan‐tissue clock. Cell‐type heterogeneity is a major confounder: each blood cell type has a distinct methylation profile, and age‐related shifts in immune composition will bias clock readings.^126^ Clocks are sensitive to acute perturbations: illness, inflammation, or even time of day can transiently alter DNAm patterns. Small procedural changes (different normalization or array version) can introduce systematic shifts, making absolute values inconsistent across studies.^127^ These sources of “noise” mean individual clock readings often have 2–3 year uncertainty, and single measurements should be interpreted cautiously.^127^, ^128^

In clinical populations like Cushing's syndrome, these limitations are magnified. Cushing's involves altered blood chemistry, chronic inflammation and corticosteroid feedback on immune cells (e.g., lymphopenia). Such factors can distort methylation‐age markers. For instance, elevated cytokines and altered leukocyte subsets in Cushing's could artificially inflate or deflate clock ages compared to healthy controls.^70^, ^127^ Likewise, TL in Cushing's patients might not follow expected age curves if hematopoiesis or stress‐induced cell turnover is affected. In summary, while TL, DNAmTL and epigenetic clocks correlate with age at the population level, each measure suffers from technical variability, tissue/cell specificity, and context‐dependence.^115^, ^126^, ^128^ These caveats limit their precision for individual subjects and for diseases like Cushing's, where endocrine dysregulation and inflammation can confound the aging signals.

Furthermore, since virtually all molecular aging measurements in CS have been performed in peripheral blood, the causal chain from glucocorticoid excess → tissue‐specific molecular aging → organ dysfunction → excess mortality remains empirically unproven. The organs most relevant to CS mortality (visceral adipose tissue, liver, vascular endothelium, and bone) divide at rates fundamentally different from leukocytes, and post‐mitotic tissues such as neurons, which are profoundly affected by hypercortisolism, would not be expected to accumulate telomere attrition through replication‐dependent mechanisms at all.^120^, ^123^ Resolving this limitation requires studies that incorporate tissue‐relevant sampling strategies alongside concurrent blood‐based biomarkers, as detailed in Section 5.

## 5 FUTURE RESEARCH DIRECTIONS

The heterogeneity of the available evidence reviewed in this manuscript, far from representing a definitive negative result, highlights fundamental methodological and conceptual gaps that future research must address to determine whether Cushing's syndrome genuinely constitutes a model of stress‐accelerated biological aging. Several specific research priorities emerge from this critical appraisal.

### 5.1 Longitudinal designs with standardized multi‐platform telomere measurement

A central limitation across existing CS telomere studies is their predominantly cross‐sectional nature and reliance on a single measurement platform.^55^, ^56^, ^57^, ^58^, ^59^ Future investigations should adopt prospective longitudinal designs that follow patients from diagnosis through treatment and long‐term remission, incorporating orthogonal TL assays in parallel, specifically TRF as the reference standard alongside qPCR and DNAmTL, to enable direct methodological comparison within the same cohort.^60^, ^111^ Given that DNAmTL has demonstrated stronger prediction of all‐cause mortality, frailty, and cancer risk compared to direct TL assays in several large cohorts,^12^, ^109^ its systematic inclusion in CS studies is particularly warranted. Crucially, leukocyte cell sorting prior to TL measurement should be performed to correct for GC‐induced shifts in lymphocyte‐to‐neutrophil ratios, which represent a major uncontrolled confound in all existing adult CS studies.^60^, ^61^

### 5.2 Integration of epigenetic clocks and pace‐of‐aging estimators

No published study in CS has yet applied second‐generation epigenetic clocks, such as GrimAge, GrimAge2, or PhenoAge, to directly quantify biological age acceleration in active versus remitted hypercortisolism.^106^, ^107^ Given that each 5‐year epigenetic age acceleration has been associated with appreciable increases in all‐cause mortality risk at the population level,^104^, ^108^ applying these tools to well‐characterized CS cohorts before and after treatment would allow a direct test of whether hypercortisolism accelerates the methylation‐based biological clock. Pace‐of‐aging clocks such as DunedinPACE, which measure the *rate* of biological aging rather than cumulative age, may be particularly informative in CS, where the disease has a defined onset and can be cured, offering a unique before‐after model unavailable in most aging research.^113^, ^114^

### 5.3 Multi‐tissue and tissue‐relevant sampling strategies

To address the tissue‐specificity constraints detailed in Section 4, future studies should move beyond peripheral blood. Virtually all molecular aging measurements in CS have been performed in peripheral blood, a tissue whose methylation and TL dynamics may not accurately reflect organs most affected by hypercortisolism, such as adipose tissue, bone, hippocampus, and vascular endothelium.^111^ Future studies should seek to incorporate tissue‐relevant sampling where feasible, for example, adipose biopsies obtained at the time of adrenalectomy, or cerebrospinal fluid in patients undergoing pituitary surgery, and should leverage multi‐tissue epigenetic clocks that integrate methylation signals across compartments.^113^ Animal models of chronic corticosterone exposure, which allow tissue‐specific molecular aging assessment, should be used in parallel to validate blood‐based proxies against organ‐level aging endpoints.^16^, ^63^

### 5.4 Explicit modeling of metabolic and inflammatory confounders

The work of Aulinas et al.^57^ demonstrated that dyslipidemia and systemic inflammation, rather than hypercortisolism per se, were the primary predictors of TL shortening in CS. This finding, replicated in part by the association between TL and metabolic markers in the pediatric cohort of Tatsi et al.,^56^ underscores the need for future studies to explicitly model cardiometabolic comorbidities (diabetes, hypertension, dyslipidemia, and obesity) and inflammatory markers (IL‐6, CRP, and TNF‐α) as potential mediators or confounders, rather than merely adjusting for them. Mediation analyses, asking what proportion of the GC‐TL or GC‐epigenetic clock association is mediated by metabolic versus direct molecular pathways, would provide mechanistic clarity that descriptive associations cannot.^57^, ^70^

### 5.5 Causal inference and Mendelian randomization approaches

The cross‐sectional and often confounded nature of observational CS studies limits causal inference regarding whether glucocorticoid excess drives molecular aging or whether common upstream factors (e.g., inflammation, metabolic dysfunction) explain observed associations. Where sufficiently large CS registries exist, Mendelian randomization approaches leveraging genetic instruments for cortisol levels or HPA axis sensitivity could provide evidence more robust to confounding.^5^, ^15^ Similarly, natural experiments, such as comparing molecular aging trajectories in patients who achieve rapid surgical cure versus those with persistent hypercortisolism or incomplete remission, could help dissociate the reversible from the durable epigenetic effects of glucocorticoid excess.^7^, ^100^

### 5.6 CS subtype‐stratified analyses

As introduced in Section 1.2, endogenous CS arises from distinct etiologies, pituitary‐dependent Cushing's disease, adrenal adenoma or carcinoma, and ectopic ACTH syndrome, which differ in their cortisol secretory patterns, disease duration, and associated comorbidity profiles.^1^, ^2^, ^3^, ^4^ No published molecular aging study in CS has stratified analyses by disease subtype or formally tested for subtype‐specific effects on TL or epigenetic age. Given that ectopic ACTH syndrome typically produces more severe and rapid hypercortisolism than pituitary disease, and that adrenal CS may differ in circadian dysregulation patterns, subtype‐stratified designs could reveal whether the magnitude of molecular aging acceleration is proportional to the degree and tempo of cortisol excess, rather than simply its presence.^1^, ^82^

Collectively, these research priorities converge on a common methodological imperative: future studies of biological aging in CS must move beyond single‐platform, cross‐sectional, blood‐only designs toward longitudinal, multi‐biomarker, multi‐tissue investigations with rigorous confounder adjustment and adequate statistical power. Only through such methodological advances will it be possible to determine whether Cushing's syndrome truly represents an accelerated aging model, or whether its excess mortality reflects primarily metabolic and cardiovascular burden independent of molecular senescence pathways.

## 6 CONCLUSION

In this review, we propose Cushing's syndrome as a compelling “natural experiment” for investigating whether chronic stress, mediated by sustained glucocorticoid excess, accelerates biological aging through mechanisms such as telomere attrition and epigenetic remodeling. Yet, despite the intuitive appeal of this model, the empirical evidence discussed throughout the manuscript remains heterogeneous and, in many cases, inconclusive. This raises an important question: why has Cushing's syndrome failed to yield definitive answers? Rather than representing a disappointing null result, this apparent paradox may instead highlight important methodological and conceptual limitations in how stress‐related aging has been studied in human populations. Factors such as disease heterogeneity, differences in methodological approaches to measuring aging biomarkers, tissue‐specific effects, and the complex interaction between endocrine, metabolic, and circadian alterations may obscure clear biological signals. Recognizing these challenges is essential for refining the experimental framework and designing future studies capable of more definitively testing the glucocorticoid‐aging hypothesis.

Despite these open questions, the evidence reviewed here already conveys three lessons of broader significance for the field of stress biology and aging. First, the relationship between glucocorticoid excess and molecular aging biomarkers is not simple or direct: findings from Cushing's syndrome indicate that telomere shortening correlates more strongly with metabolic comorbidities (dyslipidemia and systemic inflammation) than with cortisol levels or disease duration per se, suggesting that stress hormones accelerate biological aging primarily through downstream metabolic and inflammatory cascades rather than exclusively through direct molecular clock mechanisms. Second, chronic glucocorticoid excess leaves durable epigenomic scars that persist beyond biochemical remission: genome‐wide hypomethylation, altered histone marks at metabolic gene *loci*, and dysregulated circadian clock gene expression have all been documented in patients after successful treatment, implying that even when the hormonal stressor is removed, the molecular aging trajectory does not fully reset. Third, and perhaps most actionably, Cushing's syndrome exposes a critical gap in the stress‐aging literature: no published study has yet applied second‐generation epigenetic clocks, tools that have demonstrated superior mortality prediction since 2019, to active versus remitted hypercortisolism. This omission defines the most important experiment still to be done, and Cushing's syndrome, with its defined onset, quantifiable cortisol exposure, and surgically curable course, remains the optimal human model in which to conduct it.

## AUTHOR CONTRIBUTIONS

**Israel Nunes Silveira:** writing – review and editing. **Alexandro Guterres:** conceptualization; writing – original draft; writing – review and editing.

## CONFLICT OF INTEREST STATEMENT

The authors declare no conflicts of interest.

## Data Availability

Data sharing not applicable to this article as no datasets were generated or analysed during the current study.
